# Corrigendum

**DOI:** 10.1002/ehf2.13337

**Published:** 2021-03-31

In the paper by Van Spall *et al*.^1^ The following text must be added in the Abstract section after Conclusion:

**Trial Registration Clinical:** Trials.gov Identifier: NCT02112227.
